# Post-traumatic chest wall lipoma in a violinist: fact or fiction?

**DOI:** 10.1093/icvts/ivab266

**Published:** 2021-10-14

**Authors:** Ergin Erginöz, Gökçe Hande Çavuş, Sinan Çarkman

**Affiliations:** 1 Department of General Surgery, Istanbul University Cerrahpasa—Cerrahpasa School of Medicine, Istanbul, Turkey; 2 Department of Pathology, Istanbul University Cerrahpasa—Cerrahpasa School of Medicine, Istanbul, Turkey

**Keywords:** Lipoma, Chest wall tumour, Swelling

## Abstract

Lipomas are benign soft tissue tumours that can occur anywhere on the body and are rarely encountered on the chest. The pathophysiology between soft tissue trauma and lipoma development is not fully understood, and various theories have been presented. We present the case of a violinist with a 40-year occupational history who presented with swelling of the left upper chest wall. The microscopic sample of the resected lipoma showed inflammatory cells with fat necrosis, which are features thought to be involved in the development of a lipoma following soft tissue trauma.

## INTRODUCTION

Lipomas are known to be the most common benign subcutaneous soft tissue tumours, with a prevalence of 2.1% [1]. They are tumours of the adipocytes that are encapsulated with a thin layer of fibrous tissue and can occur anywhere in the body. The differential diagnosis includes benign conditions such as sebaceous cyst, abscess or a hibernoma. However, malignant conditions such as liposarcoma should also be considered.

The pathophysiology of lipoma development is not well understood, and limited research is available showing a connection between a post-traumatic event involving the soft tissue and the development of a lipoma. We present the case of a giant chest wall lipoma and show the possible relationship between soft tissue trauma and lipoma development.

## CASE PRESENTATION

**Figure 1:**
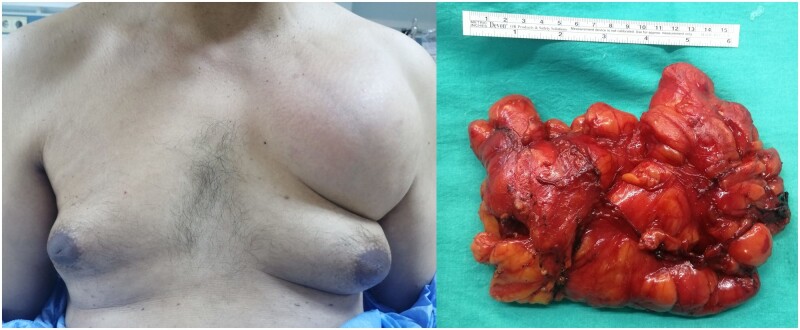
Swelling in the left upper anterior thoracic wall and a surgical specimen of a 18.5 × 13 × 7.5 cm, multilobulated mass of the left upper chest wall.

A 47-year-old male patient presented to the clinic with a slowly growing, chronic swelling of nearly 10 years in the left upper chest region (Fig. 1). His medical history was unremarkable except for a surgical intervention for cervical stenosis in 2019. His family history included a lipoma in the extremities in both uncles. The patient’s occupation included a 40-year history of playing the violin. Physical examination revealed a non-tender, fixed swelling in the left upper chest nearly 20 cm × 15 cm. The laboratory parameters were normal. A non-contrast chest computed tomography scan did not reveal any invasion of the mass into the thoracic wall. An operation was performed to alleviate discomfort and for cosmetic reasons.

**Figure 2:**
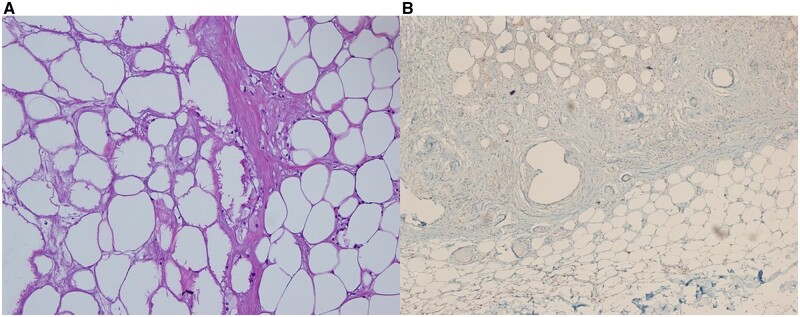
(**A**) Fat necrosis and the accompanying sparse lymphocytes in the lipoma (haematoxylin and eosin stain with 40× magnification). (**B**) Adipocytes are negative for immunohistochemistry with CDK4.

After the resection, the pathological report was consistent with a lipoma (Fig. 1). Immunohistochemical staining was negative for cyclin-dependent kinase 4, indicating a benign condition and ruling out malignancy (Fig. 2).

## DISCUSSION

Subcutaneous lipomas are common benign soft tissue tumours that can occur anywhere on the body, but chest wall lipomas are rarely encountered. They are usually located superficially; however, intramuscular and intrathoracic localizations can also be observed. Lipomas are often harmless and are usually removed if they cause pain or for cosmetic reasons. Lipomas deeply situated in the chest wall may cause organ dysfunction and interfere with heart and lung functions due to compressive effects, in which case surgery is warranted [1].

Genetic factors have been shown to play a role in the development of a lipoma. Nearly two-thirds of patients display genetic alterations where the HMGA2 gene and 12q13-15 chromosomal aberrations are shown to be involved in the pathogenesis of the tumour [2]. Gardner syndrome, Madelung disease, multiple hereditary lipomatosis and adiposis dolorosa are some of the genetic disorders associated with the development of lipomas [2].

The mechanism of post-traumatic lipoma development is not fully understood, but several theories exist that support this formation. One hypothesis is that they develop due to prolapse of the adipocytes, so-called pseudolipomas, as a result of blunt soft tissue trauma [3]. The other hypothesis is based on the effect of cytokines and growth factors released by the local inflamed tissue after blunt soft tissue trauma, which are thought to cause differentiation of preadipocytes into mature adipocytes [2, 3]. Trauma can result in necrosis in adipose tissue and result in local inflammation, which may then lead to lipoma formation [2].

A common cutaneous lesion for violin and viola players is fiddler’s neck, which is a type of trauma-induced dermatitis on the left side of the neck, below the jaw. Various studies in the literature show a relationship between trauma and soft tissue tumour development. Signorini and Campiglio [4] presented a series of 9 patients in whom a subcutaneous lipoma developed several months after blunt trauma. In a series of 10 patients with lipoma formation secondary to blunt trauma, Copcu and Sivrioglu [5] offered an inflammatory-based explanation of the mechanism of post-traumatic lipoma development whereby patients after trauma developed a long-lasting bruising that was found to be related to fat necrosis.

This case is unique given that the 40-year history of playing the violin posed a risk for the patient by causing chronic irritation and trauma and leading to the development of a lipoma. Further, inflammatory cells and fat necrosis were observed in the specimen, which are microscopic features listed as possible mechanisms of lipoma development following trauma.

**Conflict of interest:** none declared. 

### Reviewer information

Interactive CardioVascular and Thoracic Surgery thanks Phillip Antippa, Joao-Carlos Das-Neves-Pereira and the other anonymous reviewers for their contribution to the peer review process of this article.
